# Systemic Effects of Repeated Intraperitoneal Application of Graphene Oxide and Polyethylene Glycol-Functionalized Graphene Oxide Nanoparticles in Long Evans Male Rats

**DOI:** 10.3390/ijms27125522

**Published:** 2026-06-18

**Authors:** Milena Keremidarska-Markova, Bilyana Ilieva, Dilyana Doncheva-Stoimenova, Milena Shkodrova, Dimitrina Atanasova, Madlena Andreeva, Desislava-Aida Badi, Kamelia Hristova-Panusheva, Trayana Kamenska, Natalia Krasteva, Mariela Chichova

**Affiliations:** 1 Faculty of Biology, Sofia University St. Kliment Ohridski, 1164 Sofia, Bulgaria; b.ilieva@biofac.uni-sofia.bg (B.I.); donchevast@biofac.uni-sofia.bg (D.D.-S.); mshkodrova@biofac.uni-sofia.bg (M.S.); 2 Centre of Competence “Sustainable Utilization of Bio-resources and Waste of Medicinal and Aromatic Plants for Innovative Bioactive Products” (BIORESOURCES BG), 1000 Sofia, Bulgaria; m.andreeva@inb.bas.bg (M.A.);; 3 Institute of Neurobiology, Bulgarian Academy of Sciences, 1113 Sofia, Bulgaria; d.atanasova@inb.bas.bg; 4 Department of Anatomy, Faculty of Medicine, Trakia University, 6000 Stara Zagora, Bulgaria; 5 Institute of Biophysics and Biomedical Engineering, Bulgarian Academy of Sciences, 1113 Sofia, Bulgaria; kpanusheva@biomed.bas.bg (K.H.-P.); trayanakamenska@abv.bg (T.K.); natalia.krasteva@yahoo.com (N.K.)

**Keywords:** graphene oxide biocompatibility, polyethylene glycol-modified graphene oxide, nanomaterials toxicity, physiological effects, subacute toxicity

## Abstract

Recently, nanosized graphene oxide (nGO) has gained significant scientific interest in biomedical strategies. However, before clinical translation, GO-based nanomaterials must be thoroughly evaluated for safety and biocompatibility. Therefore, this study investigated the in vivo effects of pristine GO and polyethylene glycol-functionalized GO (nGO-PEG) nanoparticles in male Long Evans rats, following repeated intraperitoneal administration (4 mg/kg body weight). The effects of the nanoparticles were assessed using a range of physiological and pathological markers including body weight (BW) gain, organ coefficients, diuresis, histological, hematological and biochemical parameters. Both nGO and nGO-PEG significantly suppressed BW gain and reduced diuresis in treated rats. Nanoparticle exposure resulted in significant kidney enlargement and reduced testes weight. Mild histological alterations were observed in all examined organs, with nGO showing a tendency toward slightly more pronounced changes than nGO-PEG. Serum levels of aspartate aminotransferase, alanine aminotransferase, and creatinine were significantly elevated in nGO-treated rats, whereas nGO-PEG significantly increased the urinary levels of creatinine and urea. Both nGO- and nGO-PEG-treated rats exhibited elevated serum glucose concentrations. Significant hematological changes were detected in rats treated with both nanoparticles with pronounced effects observed following nGO-PEG administration. Our results suggest possible hematological and metabolic disturbances, as well as hepatic injury and renal toxicity in rats at repeated exposure to nGO and nGO-PEG.

## 1. Introduction

The constant search for new materials capable to meet the high standards for biomedical application has recently turned the scientific interest onto graphene oxide (GO) and its derivatives [1,2]. Due to their favorable electric, thermal, mechanical and chemical properties, GO nanomaterials are promising candidates for various biomedical strategies [3]. In particular, GO-based nanomaterials have been extensively explored as platforms for targeted drug delivery, biosensing, tissue engineering, antimicrobial coatings, and photothermal or photodynamic cancer therapy [4,5,6]. The abundance of oxygen-containing functional groups on the GO surface enables efficient loading of therapeutic molecules and facilitates further chemical modification, supporting the development of multifunctional nanoplatforms for precision medicine [7].

Despite their explicit advantages, when considering GO nanomaterials for biomedical purposes, challenges like aggregation in physiological environments require some modifications to improve the biocompatibility and stability of GO in biological systems [8]. Functionalization with polyethileneglycol (PEGylation) is among the most commonly applied strategies of GO nanoparticles (NPs) enhancing aqueous solubility, drug loading capacity and reducing cytotoxicity [9,10]. Numerous in vitro studies have demonstrated that while PEGylated GO composites are effective against many cancer cell lines, they typically show minimal toxicity in normal cells, supporting their suitability for anticancer and tissue engineering applications [11,12,13,14]. These findings highlight the promise of GO-based nanomaterials for future biomedical and theranostic applications. However, it is important to note that these data are based mainly on short-term studies (≤96 h). Despite the growing number of in vitro investigations, knowledge regarding the in vivo behavior and systemic safety of GO NPs remain scarce and contradictory [15,16]. Existing animal studies report inconsistent findings concerning biodistribution, biodegradation, inflammatory responses, and organ toxicity, which may depend on factors such as particle size, oxidation degree, surface functionalization, dosage, administration routes, concentrations, treatment frequency and animal models [17]. Moreover, many available studies focus on acute or short-term exposure, whereas information regarding the consequences of repeated administration is still limited. Since biomedical applications of GO-based systems may require multiple administrations or prolonged exposure, evaluation of their subacute and long-term systemic effects is essential for successful clinical translation [18]. In this context, the present study evaluates the systemic effects of repeated intraperitoneal administration nanosized GO (nGO) and polyethylene glycol-functionalized GO (nGO-PEG) NPs in male Long Evans rats. The NPs were administered at a dose of 4 mg/kg body weight (BW) over a three-week period at 72 h intervals. Particular attention was given to assess the potential subacute toxicity through analysis of a number of physiological and pathological markers such as body weight gain, organ coefficients, diuresis, hematological and biochemical parameters, as well as histological alterations in major organs.

## 2. Results

### 2.1. Physicochemical Characteristics of nGO and nGO-PEG NPs

Following nanoparticle preparation, the physicochemical properties of both formulations were characterized using DLS and UV-Vis_NIR spectroscopy. DLS measurements showed that pristine nGO NPs possessed a negative surface charge of −61.93 mV and an average hydrodynamic diameter of 265.96 nm (Table 1). In contrast, PEGylated nGO (nGO-PEG) exhibited an increased average size of 458.26 nm and a less negative surface charge of −34.23 mV. The observed increase in hydrodynamic diameter following PEGylation is attributed to the successful adsorption and surface grafting of PEG molecules (0.35 kDa) onto the nGO surface, while the reduction in magnitude of the ζ-potential likely results from partial shielding or neutralization of negatively charged carboxyl (-COOH) groups by neutral PEG layer.

**Figure 1:**
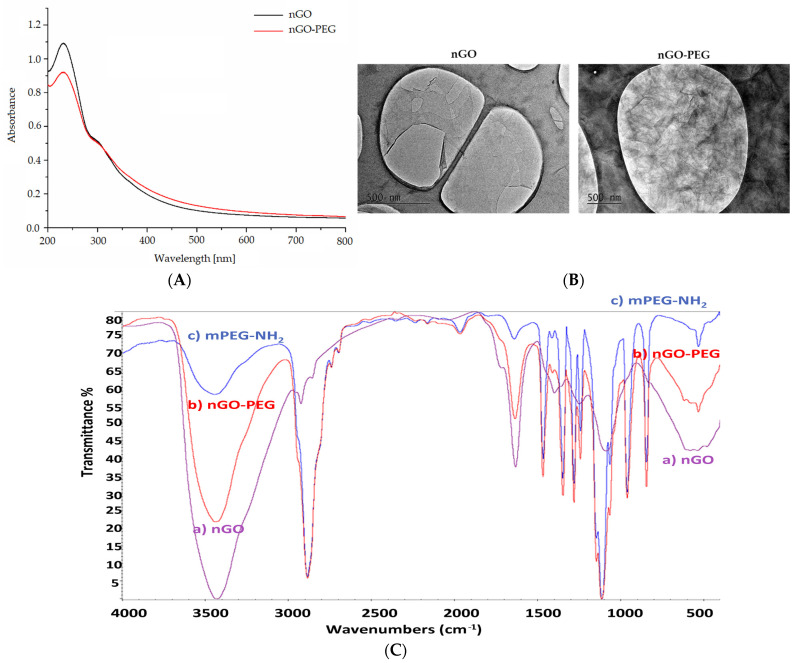
Physicochemical characterization of nGO and nGO-PEG nanoparticles. (**A**) UV–Vis absorption spectra of nGO and nGO-PEG. (**B**) TEM micrograph of nGO-PEG nanoparticles showing the characteristic morphology of PEG-functionalized graphene oxide nanosheets. (**C**) FTIR spectra of nGO and nGO-PEG. The appearance of characteristic PEG-related bands and amide-linkage vibrations confirms successful PEG conjugation to the nGO surface.

The UV-Vis spectra of both nGO and nGO-PEG displayed the characteristic absorption peak of GO at approximately 230 nm, corresponding to π→π* transitions of aromatic C=C bonds, together with a weak shoulder around 300 nm associated with n→π* transitions of C=O groups. Following PEGylation, a slight decrease in the intensity of the main absorption peak and broader absorbance at higher wavelengths were observed. This effect is consistent with partial surface coverage of nGO by PEG chains, leading to reduced exposure of optically active GO. The increased absorbance in the visible region for nGO-PEG may reflect improved colloidal stability and reduced aggregation of the NPs due to steric stabilization provided by PEG. Overall, the spectra confirm preservation of the nGO structure after PEG functionalization without disrupting the core nGO structure (Figure 1).

To further verify nanoparticle morphology and successful surface functionalization, TEM and FTIR analyses were performed. TEM analysis provides direct visualization of the nanoparticle morphology and demonstrates the structural differences between pristine nGO and PEGylated nGO, including the characteristic wrinkled morphology and partial multilayer formation observed after PEG functionalization [Figure 1B]. FTIR analysis confirmed the successful modification of GO with mPEG-NH_2_ [Figure 1C]. Compared with pristine GO, the spectrum of nGO-PEG exhibited characteristic PEG absorption bands, including CH_2_ stretching vibrations at approximately 2880–2920 cm^−1^ and a strong C–O–C ether stretching band around 1100 cm^−1^. In addition, changes in the broad O–H stretching region (~3400 cm^−1^) suggested interactions between PEG chains and oxygen-containing functional groups on the GO surface. Although distinct amide bands were not clearly observed, the appearance of PEG-specific peaks together with changes in the GO spectrum indicate successful PEG coating of the graphene oxide nanoparticles. The obtained results were consistent with our previous detailed physicochemical characterization of the same nGO and nGO-PEG nanoplatform [13,14].

Together, the UV–Vis and Zetasizer, TEM and FTIR data confirm successful PEG functionalization of nGO and demonstrate that PEGylation modifies both the surface chemistry and colloidal behavior of the NPs while preserving the characteristic electronic structure of nGO.

### 2.2. Effects of nGO and nGO-PEG on Body Weight and Diuresis

During the nanoparticle administration period, no signs of toxicity were observed and none of the animals exhibited visible physical or behavioral changes.

**Figure 2:**
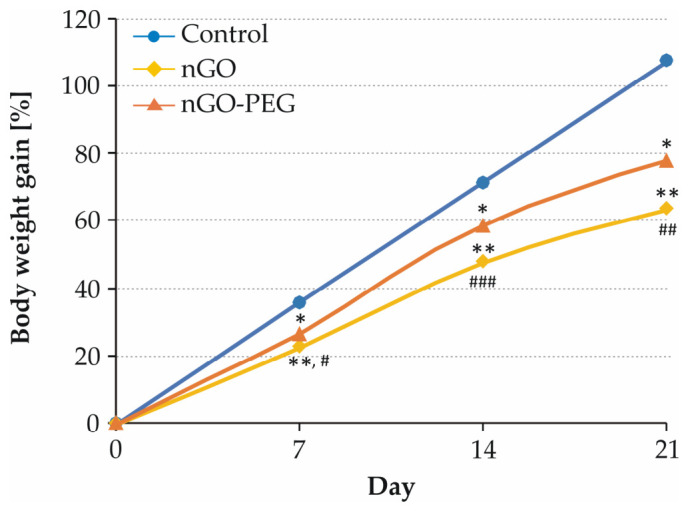
Effects of nGO and nGO-PEG nanoparticles on body weight (BW) gain over 3-week treatment period. Individual BW values were calculated as percentages of the initial BW and gain was assessed on days 7, 14, and 21 of treatment. Data are plotted as mean ± SEM of six animals. Asterisks indicate statistically significant differences compared with the control group: * *p* < 0.05, ** *p* < 0.01. Sharps indicate significant differences between the nGO and nGO-PEG-treated groups: ^#^ *p* < 0.05, ^##^ *p* < 0.01, ^###^ *p* < 0.001.

Statistical analysis indicated a significant interaction between the treatment and time, *F*(2.341, 17.554) = 19.211, *p* < 0.001, partial η^2^ = 0.72. For each of the treatment groups, the simple main effects analysis indicated statistically significant increase in the rats’ BWs during the course of the experiment compared to their initial weights (*p* < 0.001). However, post hoc analysis using Games–Howell test demonstrated that the BW gain in both nGO- and nGO-PEG-treated groups was significantly lower than that of the control group at all of the time points (Figure 2). At the same time, no changes in the daily food and water intake were observed in the treated animals throughout the experimental period.

**Figure 3:**
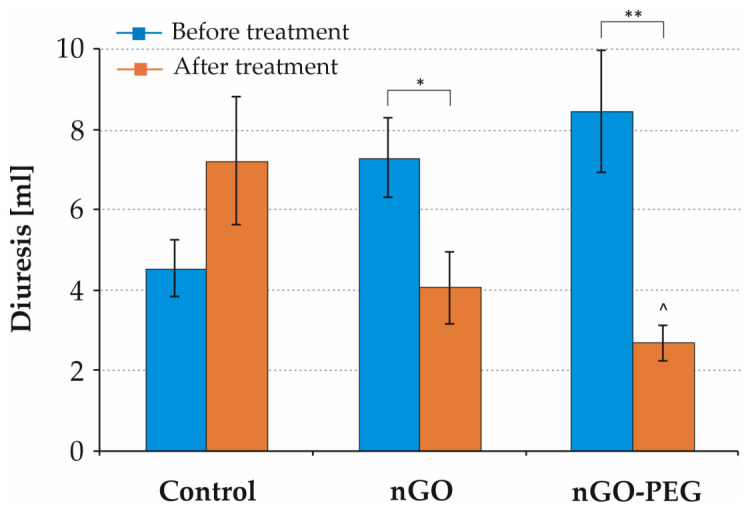
Effects of nGO and nGO-PEG on 18 h diuresis of rats measured before and after treatment. Data are plotted as mean ± SEM (n = 6 per group). Asterisks indicate statistically significant differences from the pre-treatment values: * *p* < 0.05, ** *p* < 0.01. Caret indicates significant differences between groups at specific time point: ^ *p* < 0.05.

To evaluate the effects of nGO and nGO-PEG on renal function, 18 h diuresis was measured before treatment initiation and 48 h after the final nanoparticle administration (Figure 3). At the end of the treatment period, control group showed statistically insignificant increase in diuresis, which corresponded to the increased BW (7.22 ± 1.58 mL vs. 4.53 ± 0.71 mL, *p* = 0.072). In contrast, both NP-treated groups exhibited a significant reduction in diuresis compared to values before the injections (4.07 ± 0.90 mL vs. 7.30 ± 0.99 mL, *p* = 0.034, and 2.68 ± 0.46 mL vs. 8.46 ± 1.52 mL, *p* = 0.002, respectively). Significantly lower diuresis of the nGO-PEG-treated rats was measured at the end of the experimental period compare to the corresponding value of the control animals (*p* = 0.025).

### 2.3. Histological Evaluation and Organ Indices

**Figure 4:**
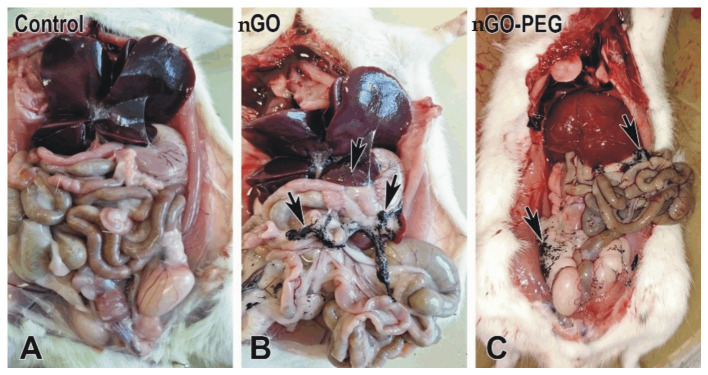
Representative macroscopic images of the abdominal cavities of rats from the control group (**A**), the graphene oxide-treated group (nGO; (**B**)), and the PEGylated graphene oxide-treated group (nGO-PEG; (**C**)) illustrating the distribution of nanoparticles within the abdominal region. Black arrows indicate visible nanoparticles aggregates.

Macroscopic evaluation of the harvested organs did not reveal any evident pathological alterations. However, a considerable accumulation of both types of NPs was observed in the abdominal cavity, particularly on and around the intestines, as well as along the mesentery surfaces and in the abdominal adipose tissue (Figure 4) while no visible agglomerates were detected in other major organs.

The mean values and standard errors of the mean (SEM) of the organ coefficients are presented in Table 2. Statistically significant effects of nanoparticle treatment were observed in kidneys and testes. The relative weight of right kidneys was significantly increased in both nGO- and nGO-PEG-treated rats compared with the control group (by 8.77% and 8.49%, respectively; *p* < 0.001), whereas a significant effect on the left kidney was observed only in the nGO-treated group (by 8.06%, *p* = 0.034). The right testes’ weight was significantly reduced in the nGO-treated group compared with the controls (by 9.46%, *p* = 0.041). Lungs of nGO-PEG-treated animals were larger compared to that of nGO-treated (by 31.54%, *p* = 0.024). No statistically significant differences were detected in the remaining organs. Although liver weight tended to be slightly greater in nanoparticle-treated animals compared with controls, these changes did not reach statistical significance.

**Figure 5:**
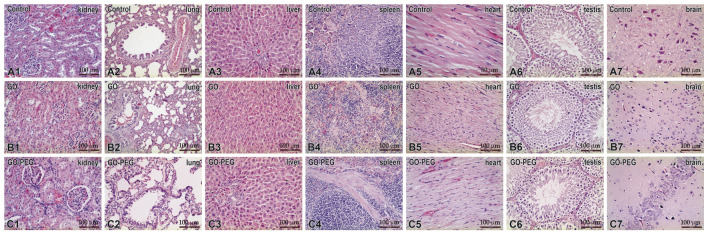
Representative light micrographs of H&E-stained histological sections from kidney, lung, liver, spleen, heart, testis, and brain in the control group (A1–A7), the nGO-treated group (B1–B7), and the nGO-PEG-treated group (C1–C7). A1/B1/C1—kidney; A2/B2/C2—lung; A3/B3/C3—liver; A4/B4/C4—spleen; A5/B5/C5—heart; A6/B6/C6—testis; A7/B7/C7—brain. Scale bars: 100 µm.

Compared with the control group, the overall tissue architecture in the nGO- and nGO-PEG-treated groups appeared largely preserved across the examined organs (Figure 5). Mild morphological differences were observed, including subtle vascular-congestive and interstitial changes, which appeared more noticeable in the kidney, lung, and spleen. In the lung, these alterations appeared somewhat more evident in the nGO group, whereas the pulmonary structure in the nGO-PEG group remained comparatively well preserved. In the liver, heart, testis, and brain, no obvious severe structural damage was observed relative to the controls. Overall, the histological changes in the treated groups were mild, with nGO showing a tendency toward slightly more apparent alterations in some organs compared with nGO-PEG.

### 2.4. Biochemical Serum and Urine Parameters and Hematology

To assess the systemic toxicity of nGO and nGO-PEG NPs, a set of biochemical parameters in serum and urine was measured (Table 3). Liver function was evaluated by measuring the serum levels of aspartate aminotransferase (AST) and alanine aminotransferase (ALT). Renal function was assessed by analysis of the serum values of blood urea nitrogen (BUN) and creatinine, as well as the urinary urea and creatinine concentrations. Serum lactate dehydrogenase (LDH) level was determined as a general marker of tissue damage and cellular injury, while creatine kinase (CK) level was measured to assess potential skeletal and heart muscle injury. Additionally, the metabolic functions were assessed by measuring the serum levels of glucose.

The serum levels of AST, ALT, and creatinine were significantly increased in the rats treated with nGO compared with the control animals (by 91.00%, *p* = 0.021; 76.25%, *p* = 0.035; 21.20%, *p* = 0.016, respectively). Also, significantly higher serum levels of glucose were measured in both nGO- and nGO-PEG-treated rats (by 87.44%, *p* = 0.019, and 176%, *p* = 0.005, respectively) in comparison to the control value. No statistically significant differences were detected in the remaining measured blood parameters. However, nGO-PEG treatment resulted in significantly increased urinary levels of creatinine and urea compared to the control (by 132.76%, *p* = 0.031, and 469.20%, *p* < 0.001, respectively). No significant changes in the calculated GFRs were observed in the treated rats compared to the control animals.

In addition, a semi-quantitative urine analysis was performed using test strips to compare the samples from the different groups, as well as to identify relative changes in the urinary parameters before and after treatment of the rats. The objective values of the parameters measured in individual animals are presented in Supplementary Table S1. Increased levels of ketones, urobilinogen, and proteins were found in most of the nGO- and nGO-PEG-treated rats compared to their corresponding pre-treatment values along with elevated leukocytes in nGO-treated group. No substantial changes were detected in the remaining urinary parameters.

The values of analyzed blood parameters are presented in Table 4. Compared to the control, nGO-PEG-treated rats showed significantly reduced white blood cell count (WBC, by 20.45%, *p* = 0.004), hemoglobin (HGB, by 16.83%, *p* = 0.014), and hematocrit (HCT, by 19.89%, *p* = 0.002), while decreased mean corpuscular hemoglobin (MCH) was determined in nGO-treated group (by 2.06%, *p* = 0.012). Both nGO and nGO-PEG decreased the mean corpuscular volume (MCV) in comparison to the control (by 5.10% and 6.82%, respectively, *p* < 0.001).

The present results were compared with the available reference values of hematological and biochemical blood parameters of male 10-week-old Long Evans RjOrl:LE rats provided by Janvier Labs (Saint-Berthevin, France) [19]. According to these reference ranges, the levels of AST and ALT of nGO-treated rats and creatinine of both treated groups exceeded the upper reference limits. Regarding hematology, values below the lower reference limits were measured for WBC, HBG, HCT, and MCV in both treated groups, as well as of RBC for nGO-PEG-treated animals. In contrast, values above the upper reference limits were registered for mean corpuscular hemoglobin concentration (MCHC) in both treated groups and for MCH in nGO-PEG-treated group. Most parameters of the control animals remained within the reference ranges, except for a slight increase in AST and creatinine, and lower WBC, platelets and HCT. These deviations from the reference values could be explained with variabilities in animal age, sampling conditions, and methodological variations during the collection of the referent data.

## 3. Discussion

The biodistribution, biotransformation, and excretion of GO can be influenced by several factors, including dose, routes of administration, physicochemical properties, particles agglomeration, surface coating, and exposure time [17]. Furthermore, properties such as the particles’ size, presence of surface functional groups, and oxygen content/surface charges may significantly affect their toxicity in biological systems [20].

The present study aimed to evaluate the biocompatibility of pristine GO and PEG-functionalized GO NPs using an in vivo model designed to assess their potential subacute toxicity following repeated intraperitoneal administration. Male Long Evans rats were exposed to the NPs at a dose of 4 mg/kg for three weeks and a range of physiological and pathological markers were analyzed.

Throughout the treatment period, none of the animals exhibited visible physical or behavioral alterations. Nevertheless, despite the absence of significant changes in the daily food and water intake, the BW gain of both nGO- and nGO-PEG-treated rats was significantly lower compared to control animals. These results are in contradiction to the data from other studies reporting no adverse effects of graphene-based nanomaterials on animal growth or body weight gain [21,22,23,24,25]. For example, Amrollahi-Sharifabadi et al. [26] observed reduced body weight gain in Wistar rats only after substantially higher GO dose (500 mg/kg) applied four times within one week. The reduced BW gain observed in the present study may reflect alterations in metabolic homeostasis induced by nanoparticle exposure. This interpretation is supported by the significantly elevated serum levels of glucose measured in both nGO- and nGO-PEG-treated rats in comparison to the control group. Moreover, increased urinary ketone levels detected in most of the treated rats further implies altered metabolism or metabolic stress, since ketone bodies are completely reabsorbed by the renal tubules at low plasma concentrations, but as plasma levels rise and the filtered load of ketone bodies increases, significant ketonuria appears [27]. Interestingly, Kurantowicz et al. [21] reported significantly lower serum levels of glucose in Wistar rats repetitively treated with GO NPs at the same dose used in the present study. Such discrepancies can be related to differences in the physicochemical characteristics of the NPs employed, since particle size and surface charge are known to strongly influence nanoparticle biodistribution, cellular interactions, and toxicity [20]. In the study by Kurantowicz et al. [21] GO NPs had an average size of 8–20 nm and a zeta potential of –8.8 mV, whereas the nGO NPs used in the current study exhibited a considerably greater average size (265.96 nm) and more negative zeta potential (–61.93 mV). These differences may substantially affect nanoparticle aggregation, tissue accumulation, and biological responses in vivo.

Further, we did not observe any signs of inflammation, necrosis, or tissue reaction near the injection sites, nor any evident organ damage was noticed. Notably, although a substantial amount of agglomerates of both types of NPs was detected within the abdominal cavity, no visible evidences of nanoparticle deposition were found in the excised organs. In agreement with these observations, no statistically significant changes were observed in the relative weights of most of the organs, with the exception of enlarged kidneys and reduced testes as the effect of nGO was more pronounced. Similarly, Abd-Alsahib and Faris [28] reported decreased testes weight in male albino rats treated with GO nanopowder at doses ranging from 20 to 60 mg/kg which was associated with deterioration of sperm formation and necrosis of the tubular epithelium. Other studies have demonstrated prolonged retention of intraperitoneally administered GO and PEGylated GO derivatives in mice accompanied by alterations in liver, brain, kidney, and spleen organ indices [15,21,29]. Kurantowicz et al. [21] also reported persistence of intraperitoneally injected GO NPs (8–25 nm) in Wistar rats, with largest agglomerates (up to 10 mm in diameter) remaining near the injection site, while smaller aggregates (~2 mm) were lodged among the mesentery and in the connective and lipid tissue in the proximity of the liver and spleen serosa. Importantly, no aggregates were detected in the kidneys, suggesting limited penetration of larger agglomerates into retroperitoneal organs through adventitia. These findings support our conclusion that intraperitoneally administered NPs primarily remain within the peritoneal cavity and associated mesenteric tissues, while systemic distribution is likely limited to sufficiently small fractions. In contratst, De et al. [30] found predominant accumulation in the liver upon 24 h of exposure of Sprague–Dawley rats to GO at a dose of 10 mg/kg, using particles with lateral dimensions below 300 nm—a size range closer to that of the nGO particles, used in the present study. This further highlights the critical role of nanoparticle size in determining systemic distribution and organ accessibility following intraperitoneal administration. Previous studies have demonstrated that GO nanosheets with diameters of 10–30 nm accumulate mainly in the liver and spleen, whereas larger sheets ranging from 10 to 800 nm tend to localize predominantly in the lungs [31,32]. Small GO nanosheets may undergo rapid renal clearance without obvious toxicity while larger NPs, such as those used in the present study (average particle size of 261.7 nm for nGO and 482.7 nm for nGO-PEG) are more prone to aggregation and sequestration by the mononuclear phagocyte system, thereby increasing the risk of toxicity [16]. In addition to particle size, the route of administration is another important factor influencing nanoparticle biodistribution and toxicological profile. Intravenously injected NPs rapidly spread throughout the circulatory system and subsequently distributed to various organs, whereas intraperitoneal administration results in slower translocation and accumulation in the organs due to gradual absorption from the abdominal cavity [29].

In the present study, histological examination revealed only mild morphological alterations primarily characterized by vascular-congestive and interstitial changes, more evident in the kidney, lung, and spleen of the GO-treated animals. Similar findings have been reported in previous in vivo studies demonstrating inflammation and more severe structural damage in animal organs such as the lung, liver, kidney, and spleen following exposure to of GO nanomaterials [23,25,33]. Interestingly, Yang et al. [15] observed substantial accumulation of PEGyalted GO derivatives, but not pristine GO, within the reticuloendothelial (RES) system, including the liver and spleen, after intraperitoneal administration in balb/c mice. The authors attributed this difference to the poor colloidal stability of unmodified GO at physiological conditions, leading to rapid aggregation after injection and consequently limiting systemic adsorption. In contrast, PEGylated GO exhibits improved dispersity and stability, facilitating gradual adsorption and accumulation in the RES organs [15,29,34]. The possible balance between the gradual adsorption from the abdominal cavity into the liver, and the biliary excretion of nanomaterials from the liver [35,36] can keep the uptake of PEGylated GO in RES organs at high levels over a long period of time, especially when high doses are applied [15].

To further assess systemic toxicity of nGO and nGO-PEG NPs, a set of biochemical serum and urinary parameters was analyzed. Evaluation of potential hepatotoxicity of graphene materials is particularly important for graphene-based nanomaterials because the liver represents the primary organ responsible for xenobiotic metabolism and detoxification [37,38,39]. Aminotransferases (AST and ALT) are widely recognized as sensitive indicators of liver damage and hepatocellular necrosis [40] as their elevation is directly proportional to the hepatic injury [41]. In the present study, repeated administration of nGO resulted in significantly elevated AST and ALT levels whereas no significant alterations were detected in nGO-PEG-treated animals. These findings indicate possible pristine nGO-induced hepatic injury, which is in accordance with previous studies. Elevated transaminase levels after GO exposure have been reported in several animal models. Rhazouani et al. [25] observed altered AST and ALT activities in male Swiss mice following prolonged repetitive administration of GO at doses of 2 and 5 mg/kg. Similarly, Nirmal et al. [33] demonstrated significant increases in AST and ALT in adult Wistar rats treated with high-dose GO (10 mg/kg), while lower doses (0.4 and 2.0 mg/kg) produced minimal effects. Acute exposure to GO at 10 mg/kg also induced elevation in ALT and AST levels in Sprague–Dawley rats [30]. On the contrary, some studies using comparable GO doses reported no significant alterations in aminotransferase levels [21,42], emphasizing the influence of a combination of factors such as particle size, surface chemistry, aggregation state, and exposure protocol on GO toxicity. In addition, our results suggest that PEGylation attenuates the hepatotoxic potential of pristine GO NPs and are in accordance with the study by Yang et al. [15] who reported no obvious hepatic toxicity in mice treated with PEGylated GO derivatives at a dose of 50 mg/kg. Also, elevated serum creatinine levels observed in the nGO-treated group further suggest possible renal impairment [16]. These indices were effectively alleviated by the PEG coating of GO. Our results also showed increased serum levels of creatinine in the GO-treated rats compared to the control animals, indicating renal function deterioration. Blood urea nitrogen (BUN) and serum creatinine are considered reliable markers of renal function [30,43,44]. Creatinine is the end product of muscle creatine breakdown and excreted by glomerular filtration in kidney. Increased level of serum creatinine indicates acute renal toxicity [30,45]. Although nGO-PEG treatment did not significantly increase the serum levels of creatinine and BUN, it induced notable elevation of the urinary creatinine and urea levels, which may also reflect renal functional disturbances. Similar increase in urinary excretion of creatinine has been reported in models of acute organophosphate intoxication [46]. Studies on the toxicity of GO on the kidneys have shown contradictory results [17]. Several studies have demonstrated significantly increased serum creatinine and BUN levels, accompanied by morphological alterations of kidney after GO or graphene administration in rats, indicating nephrotoxicity [24,30,47]. Conversely, other investigations reported no significant biochemical or structural kidney abnormalities after GO treatment [42,48,49]. In the present study, the comparison with reference values for 10-week old male Long Evans rats provided by Janvier Labs (Saint-Berthevin, France) RjOrl:LE rats [19] confirmed elevated levels of AST, ALT and creatinine of nGO-treated rats, further supporting the presence of hepatic and renal toxicity induced by repeated exposure to pristine GO NPs.

In the present study, diuresis was significantly reduced in both nanoparticle-treated groups at the end of the treatment compared to the pre-treated values. Moreover, nGO-PEG-treated rats exhibited significantly lower diuresis compared to the control animals. These findings are further supported by the semi-quantitative urine analysis which revealed treatment-associated alterations in urinary parameters including elevated levels of ketones, urobilinogen, and proteins in both NPs-treated groups, as well as increased leukocytes number in the urine of nGO-treated animals. Urobilinogen is a colorless end product of bilirubin metabolism, and increased levels of urinary urobilinogen are commonly associated with hemolytic disease and impaired liver function [50]. Proteinuria is a key indicator of kidney damage and nephrotoxicity reflecting alterations in glomerular capillary permeability and/or in tubular reabsorption or in significant increase in serum immunoglobulins [51]. Urinary proteins can be used as markers of glomerular damage, often linked to conditions like diabetes and immune disorders [52], while increased urinary concentration of low molecular weight proteins can signal tubular damage or overload [53]. In our study, proteinuria was detected even prior to treatment in all groups—a phenomenon commonly observed in rats [54,55]. However, enhanced proteinuria may indicate an early onset of glomerular dysfunction and nephrotoxicity, preceding histopathological damage [56,57].

In our study, no statistically significant alterations were observed in the serum LDH and CK levels, which are commonly used markers of tissue damage and skeletal and cardiac muscle impairment. In contrast, Aguado-Henche et al. [42] reported significantly elevated LDH levels in Wistar rats following exposure to GO at dose of 4 mg/kg, suggesting that toxic responses may vary depending on nanoparticle characteristics and experimental conditions.

Blood circulation plays a central role in the systemic distribution of carbon-based nanomaterials, and simultaneously represents the first biological barrier encountered after absorption [21]. However, available data regarding the side effects of graphene-based nanomaterials on blood cells remain inconsistent. Some authors reported minimal or no significant alterations in hematological parameters following graphene exposure [21,22,23,24,26,58,59]. Similarly, Qu et al. [60] also reported no significant change in peripheral blood indexes following a single GO administration which they attributed in part to rapid clearance by mononuclear phagocyte system. In contrast, the present study demonstrated significant hematological alterations, particularly in nGO-PEG-treated rats, including reduced WBC, HGB, hematocrit, and MCV, as well as decreased MCV and MCH values in the nGO-treated group. These findings suggest possible adverse effect on the hematopoietic and immune functions. The reduction in HGB, HCT, and MCV are signs of anemia, with possible microcytosis (smaller red blood cells). Similar findings have been previously reported by Aguado-Henche et al. [42], who observed smaller red blood cells with higher hemoglobin content in GO-treated Wistar rats, potentially representing a compensatory adaptation to reduced cell size [61]. Syama et al. [29] also reported transient decreases in RBC, HGB and HCT in mice 7 days after exposure to PEGylated GO, whereas Abd-Alsahib and Faris [28] observed reductions in the number of red blood cells and hemoglobin levels in GO-treated rats, suggesting that the sharp edges of graphene materials may mechanically disrupt erythrocyte membranes and induce hemolysis. Graphene oxide has been shown to interact directly with cellular membranes altering membrane integrity and permeability [62,63]. Although PEGylation is generally applied to improve colloidal stability and reduce nanomaterial toxicity [15,63], the present results suggest that PEGylated GO may exert stronger hematological effects than pristine GO under the tested conditions. The decrease in WBC in nGO-PEG group may reflect altered immune homeostasis, possibly resulting from sequestration of leukocytes associated with inflammatory responses. Similar leukocyte alterations have been reported following intraperitoneal administration of GO in rats [28,42] and recent studies also demonstrated transient leukopenia and changes in leukocyte subpopulations after intravenous administration of GO in mice [64]. Importantly, comparison with reference hematological intervals for male Long Evans rats [19] revealed that WBC, HBG, HCT, and MCV values in both treated groups, as well as RBC in nGO-PEG-treated animals, were below the physiological reference range. In contrast, MCHC values exceeded the upper reference limit in both nanoparticle-treated groups. These findings further support the presence of nanoparticle-induced disturbances affecting hematopoietic and immune system. Nevertheless, it should be emphasized that hematological reference ranges in laboratory animals may vary depending on sex, age, strain, and analytical methodology [22]. Moreover, there is a lack of concrete data concerning many of the biochemical parameters, especially for particular strains, including Long Evans strain.

PEGylation is generally considered advantageous for biomedical applications because it prolongs blood circulation time and reduces macrophage uptake, thereby improving nanoparticle bioavailability for imaging and drug delivery applications [65]. However, prolonged blood circulation and delayed clearance may also increase the likelihood of long-term adverse effects, underscoring the importance of comprehensive investigation of their toxicity. Previous studies demonstrated that PEG coating can reduce GO retention in organs such as the liver, lung, and spleen, promote NP clearance from these organs, and alleviate nanoscale GO-induced acute tissue injuries and fibrosis [16]. Yang et al. [34] reported no appreciable toxicity of PEGylated nanographene sheets in mice at a dose of 20 mg/kg up to 3 months after intravenous administration. In contrast, other studies have shown that PEGylated reduced GO may still induce inflammatory responses in liver, spleen, kidney and brain and changes in clinical parameters, indicating that surface functionalization does not completely eliminate graphene toxicity [29].

An important consideration in the present study is the relatively low molecular weight of the PEG used for functionalization (0.35 kDa) which is substantially lower than the 2–5 kDa PEG chains typically required to generate a dense steric barrier capable of effectively preventing protein adsorption and immune recognition [66]. Short PEG chains may partially reduce surface charge and modify aggregation behavior but are insufficient to establish a stable “stealth” coating. Although the less negative zeta potential of nGO-PEG (−34.23 mV) compared to nGO (−61.93 mV) could theoretically reduce opsonization after administration. under intraperitoneal administration, the reduced surface charge may instead favor interactions with extracellular matrix components and promote particle agglomeration and local retention within the peritoneal cavity.

Overall, both nGO and nGO-PEG induced measurable toxic effects following repeated intraperitoneal administration. Pristine nGO predominantly affected hepatic parameters, whereas nGO-PEG induced more pronounced renal and hematological alterations. These findings indicate that PEGylation does not necessarily eliminate the toxicity of GO NPs and that factors such as the route of administration, nanoparticle size, surface charge, and PEG chain length critically influence the in vivo behavior and biological effects of graphene-based nanomaterials.

## 4. Materials and Methods

### 4.1. Preparation and Characterization of nGO and nGO-PEG

GO particles were purchased as an aqueous solution with a concentration of 4 mg/mL form Graphenea (San Sebastián, Spain). To obtain nanosized GO, the commercially available GO solution was diluted to a final concentration of 2 mg/mL and sonicated for 2 h at 500 W using an ultrasonic homogenizer (VCX 500, Sonics & Materials Inc., Newtown, CT, USA). The PEGylation of nGO was performed using mPEG-NH_2_ according to a previously established protocol [13]. Briefly, GO NPs were mixed with mPEG-NH_2_ (Abbexa Ltd., Cambridge, UK), sonicated for 5 min, and incubated overnight at 70 °C in a water bath. The resulting nGO-PEG suspension was then centrifuged at 13,000× *g* for 20 min to remove unstable aggregates and stored at 4 °C. Immediately prior to animal treatment, the nanoparticle suspensions were sonicated for 1 h in an ultrasonic water bath (50 Hz, UST2.4-150, Siel, Bulgaria).

Immediately after preparation the physicochemical properties of nGO and nGO-PEG were determined. The hydrodynamic particle size and zeta potential were measured using dynamic light scattering (DLS) with a Zetasizer instrument (Malvern Instruments Ltd., Worcestershire, UK). Also, the absorption spectra of both NPs in the UV-Vis and NIR regions were recorded using a spectrophotometer (Specord 210 Plus, Edition 2010, Analytik Jena AG, Jena, Germany). All DLS measurements were performed in triplicate, and the intensity-weighted mean hydrodynamic diameter (Z-average) is reported.

The morphology of nGO and nGO-PEG NPs was examined by transmission electron microscopy (TEM; JEM-2100, JEOL, Tokyo, Japan). Prior to analysis, the nanoparticle suspensions were dispersed in deionized water and sonicated in a water bath for 60 min. A drop of each suspension was deposited onto a carbon-coated copper grid, air-dried at room temperature, and subsequently imaged at an accelerating voltage of 200 kV.

Fourier transform infrared (FTIR) spectroscopy was performed to evaluate the surface functional groups and confirm PEGylation. The spectra of dry nGO and nGO-PEG samples were recorded using a Thermo Nicolet 6700 spectrometer (Thermo Fisher Scientific, Waltham, MA, USA) in the mid-infrared region (4000–400 cm^−1^) at a spectral resolution of 2 cm^−1^.

### 4.2. Animals

In the present study, 18 male Long Evans rats (5 weeks old, 131.94 ± 3.78 g BW) were used. Female animals were excluded from the experimental design to avoid possible cyclic hormonal variations that could increase experimental variables and affect the interpretation of the results. The animals were obtained from the Vivarium with Physiological Laboratory at the Centre of Competence “Sustainable Utilization of Bio-resources and Waste of Medicinal and Aromatic Plants for Innovative Bioactive Products” (BIORESOURCES BG), project BG16RFPR002-1.014-0001, at the Faculty of Biology of Sofia University “St. Kliment Ohridski”.

The animals were housed in polycarbonate cages with stainless steel wire tops, with six animals per cage, and were provided a standard pelleted diet (TopMix^®^ Laboratory Animals, HL-TopMix Ltd., Sliven, Bulgaria) and water ad libitum. Housing conditions were maintained at 22 ± 2 °C, 50–60% humidity, and 12:12 h light–dark cycle. During the experimental period, the animal behavior and health conditions were monitored.

Efforts were made to minimize the number of animals used and to reduce their suffering. All experimental procedures were strictly performed in accordance with the regulations outlined in Directive 2010/63/EU of the European Parliament and of the Council of 22 September 2010 regarding the protection of animals used for scientific purposes. The study was approved by the Bulgarian Food Safety Agency under the Ministry of Agriculture, Food and Forestry (Permit No. 381/12.03.2024).

### 4.3. Experimental Protocol and Groups

The rats were randomly divided into three groups (n = 6 per group): control, nGO- and nGO-PEG-treated groups, respectively. Nanoparticle suspensions with a concentration of 2 mg/mL were administered intraperitoneally at a dosage of 4 mg/kg BW over three weeks at 72 h intervals (eight injections in total). Animals in the control group received equivalent volumes of vehicle (sterile distilled water) following the same administration schedule. Observations of food and water intake were daily performed during the whole experimental period.

**Figure 6:**
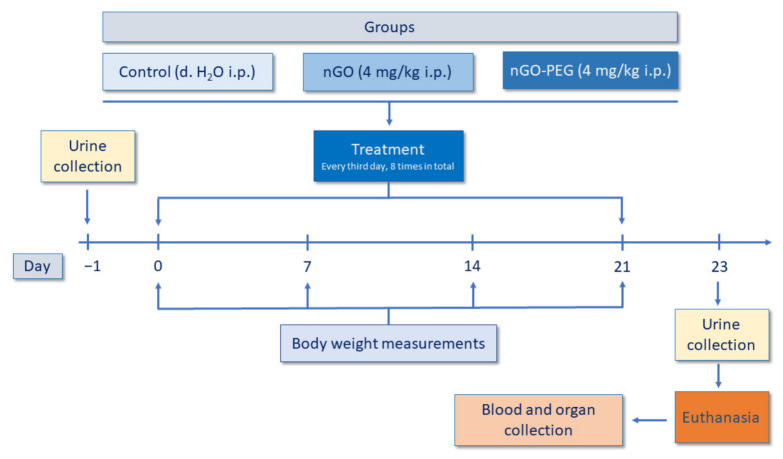
Diagram of the experimental design. i.p.—intraperitoneally.

The overall experimental design is presented in Figure 6.

Two days after the final dose, the animals were euthanized by intraperitoneal administration of Ketamine 100 mg/kg (Ketamine hydrochloride, Anaket, 100 mg/mL injectable solution, Vetviva Richter GmbH) and Xilazin 10 mg/kg (Xylazin 2%—inj.ad.us.vet, Bioveta, a. s., Ivanovice na Hané, Czech Republic), and blood and organ samples were collected for further evaluations.

### 4.4. Body Weight Changes, Organ Indices, and Diuresis

Body weight of each animal was measured at the beginning of the experiment (day 0) and on days 7, 14, and 21 of treatment. BW gain was expressed as a percentage of the initial body weight. Mean BW values for each group were plotted over time to illustrate growth dynamics throughout the experimental period.

At the end of the study, brain, liver, heart, kidneys, spleen, lungs, and testis were excised and weighed. Relative organ weights (organ coefficients) were calculated based on final BW using the following formula:

organ coefficient (g/100 g) = (organ weight/rat BW) × 100

Urine samples were collected from each rat twice during the experimental period—prior to treatment initiation (day −1) and 2 days after the final injection—by placing the animals in individual metabolic cages with free access to water and food deprivation for 18 h. Immediately after collection, urine was analyzed for leukocytes, ketone, urobilinogen, bilirubin, protein, glucose, and pH by using BM URI 10 test strips (BioMaxima S.A., Lublin, Poland) and a semi-automated BM URI 200 urine analyzer (BioMaxima S.A., Lublin, Poland). In addition, urinal creatinine and urea levels were estimated as described in Section 4.6.

### 4.5. Histological Tissue Processing and Evaluation

At the end of the experiment, the kidney, lung, liver, spleen, heart, testis, and brain were carefully dissected and collected. The obtained tissue samples were placed in 10% buffered formalin for fixation to ensure preservation of tissue architecture and cellular detail. After fixation, the samples were subjected to standard histological processing and carried through to paraffin embedding. Serial sections with a thickness of 6 µm were prepared from the resulting paraffin blocks. The sections were mounted on glass slides and stained with hematoxylin and eosin (H&E) routinely. The prepared specimens were then examined under light microscopy and compared to assess the histological changes in the studied organs. The prepared slides were observed with a Leica DM1000 research microscope and a Leica DFC 290 digital camera (Leica Microsystems, Wetzlar, Germany). For better visualization of the structures under investigation, the contrast of the images was enhanced in Adobe Photoshop 24.1.0. The morphological alterations in the organs under study were determined by examining the stained tissue sections under the light microscope (Leica Microsystems, Wetzlar, Germany).

Photographs of every rat’s insides were taken immediately after sacrifice.

### 4.6. Hematological and Biochemical Analyses

Blood samples from control rats and rats treated with nGO- and nGO-PEGwere collected through a cardiac puncture at the time of their sacrifice. Each blood sample was divided into two aliquots. The first aliquot was placed in BD Vacutainer^®^ blood collection tubes containing K3-EDTA (Becton, Dickinson and Company, Plymouth, UK) for hematological analysis and processed within 6 h. The following hematological parameters in whole blood were analyzed using Mindray BC-6800 automatic hematology analyzer (Mindray, Shenzhen, China): WBC, RBC, HGB, HCT, MCV, MCH, MCHC, and PLT.

The second blood aliquot was collected for serum preparation in BD Vacutainer^®^ SST^TM^ tubes containing gel and clot activator (Becton, Dickinson and Company, Plymouth, UK). The blood was allowed to clot thoroughly for 60 min and centrifuged at 1300× *g* for 15 min at 25 °C. The serum biochemical assay included AST, ALT, LDH, creatinine, BUN, glucose, and CK. These parameters were analyzed using a semi-auto chemistry analyzer URIT—880 Vet (URIT Medical Electronic Co., Ltd., Guangxi, China) with standard diagnostic kits (BioSystems S.A., Barcelona, Spain) following the manufacturer’s protocol. In addition, the levels of urine creatinine and urea were also measured using the same methodology. Further, GFR was determined by calculation of creatinine clearance (CrCl) using the formula:

$$CrCl\;\left[ml\times {min}^{-1}\times 100\;g\;{BW}^{-1}\right]=\frac{U_{Cr}\times V_{u}\times 100}{P_{Cr}\times t\times BW},\;$$

where *U_Cr_* and *P_Cr_* are the urine and plasma concentrations of creatinine, respectively, *V_u_* is the volume of urine collected over the time *t*, and BW is the rat final body weight measured at 21st day of the treatment.

### 4.7. Statistical Analysis

The data obtained are presented as mean values ± standard error of the mean (SEM). Changes in the studied parameters were calculated as percentage changes compared to the values of the corresponding control or treated groups. Normality of the dependent variables was assessed using the Shapiro–Wilk test. Levene’s test was used to assess the equality of error variances. A One-way Analysis of Variance (ANOVA) followed by post hoc analysis with a Tukey test was conducted to explore the impact of the treatment on the organ coefficients and biochemical and hematological parameters. When the assumption of equal variances was violated, Wellch’s ANOVA test followed by Games–Howell post hoc analysis was used. The data from some of the groups were non-normally distributed, therefore the non-parametric Kruskall–Wallis H test was employed followed by Dunn’s post hoc test with Bonferroni correction. The effects of the treatment and time on the BW gain and diuresis were evaluated by a One-way Repeated Measures ANOVA. Mauchly’s test indicated that the assumption of sphericity had been violated for the BW gain data; therefore, Greenhouse–Geisser corrected results are reported. Significant interactions between the two factors, treatment and time, were observed; therefore, Simple Effects Analysis was performed as follows. To compare the effect of the treatment at the different time points, Univariate ANOVA test was conducted followed by Tukey post hoc analysis for the diuresis data and Games–Howell post hoc test for the BW gain data respectively. A Repeated Measures ANOVA with Bonferroni adjustment for multiple comparisons was used to evaluate the effect of time in each of the groups. A value of *p* < 0.05 was considered significant. All statistical analyses were computed using IBM^®^ SPSS^®^ Statistics version 27.

## 5. Conclusions

The present study demonstrated that repeated intraperitoneal application of nGO and nGO-PEG at a dose of 4 mg/kg induced pronounced metabolic disturbances as evidenced by reduced body weight gain and elevated serum glucose levels. Decreased diuresis, together with increased serum levels of aminotransferases and creatinine in nGO-treated rats, and elevated urinary creatinine and urea levels in the nGO-PEG group, suggest pathological alterations in the liver and kidney functions. Furthermore, a significant impact of the NPs on the hematological parameters was observed in both treated groups, indicating possible disturbances of the hemopoetic and immune systems. Although only mild histological alterations were detected, the observed biochemical and hematological changes may represent early indicators of subclinical or “silent” toxicity. Importantly, PEG functionalization did not fully mitigate the adverse effects associated with GO exposure under the present experimental conditions. These findings emphasize the necessity for careful evaluation of the long-term in vivo safety of GO-based nanomaterials prior to their broader biomedical application and clinical translation.

## Figures and Tables

**Table 1:** The average hydrodynamic size and zeta potential of nGO and nGO-PEG.

Parameters/Samples	nGO	nGO-PEG
Average particle size [nm]	265.96 ± 3.23	458.26 ± 13.34
Surface charge [mV]	−61.93 ± 0.55	−34.23 ± 0.177

**Table 2:** Mean relative organ weights (g/100 g body weight) of the control group and groups treated with nGO and nGO-PEG nanoparticles.

Organ	Organ Coefficient, g/100 g		
	Control	nGO	nGO-PEG
Brain	0.759 ± 0.015	0.791 ± 0.013	0.766 ± 0.009
Liver	3.178 ± 0.079	3.417 ± 0.127	3.561 ± 0.119
Heart	0.432 ± 0.041	0.356 ± 0.016	0.369 ± 0.009
Kidney left	0.388 ± 0.007	0.419 ± 0.008 *	0.412 ± 0.008
Kidney right	0.412 ± 0.005	0.449 ± 0.007 ***	0.447 ± 0.004 ***
Spleen	0.238 ± 0.008	0.242 ± 0.010	0.269 ± 0.009
Lung	0.587 ± 0.044	0.488 ± 0.036	0.641 ± 0.031 ^#^
Testis left	0.501 ± 0.023	0.447 ± 0.011	0.454 ± 0.009
Testis right	0.479 ± 0.012	0.434 ± 0.011 *	0.442 ± 0.012

Organ coefficients were calculated based on the final BW measured on day 21 of treatment. Data are presented as mean ± SEM (n = 6 per group). Asterisks indicate significant differences compared with the control group: * *p* < 0.05, *** *p* < 0.001. Sharps indicate significant differences between the nGO and nGO-PEG-treated groups: ^#^ *p* < 0.05.

**Table 3:** Biochemical serum and urine parameters of the control group and groups treated with nGO and nGO-PEG nanoparticles.

Parameter	Reference Range ^1^	Control	nGO	nGO-PEG
**Serum:**				
AST, U/L	129.0 ± 25.0	200.98 ± 30.92	383.88 ± 62.85 *	156.89 ± 29.97 ^##^
ALT, U/L	67.0 ± 9.0	44.95 ± 3.44	79.23 ± 14.66 *	45.42 ± 11.97
LDH, U/L	N.A.	1711.19 ± 332.71	1928.62 ± 233.95	1112.59 ± 269.86
CK, U/L	N.A.	789.03 ± 243.23	1780.41 ± 281.34	1695.55 ± 179.90
Glucose, mg/dL	290.0 ± 70.0	106.45 ± 7.53	199.53 ± 22.88 *	293.97 ± 33.35 **
Creatinine, mg/dL	0.51 ± 0.03	0.78 ± 0.03	0.94 ± 0.02 *	1.18 ± 0.27
BUN, mg/dL	N.A.	19.71 ± 1.60	21.15 ± 2.08	18.04 ± 4.50
**Urine:**				
Creatinine, mg/dL	N.A.	69.87 ± 18.08	131.91 ± 12.88	162.64 ± 33.29 *
Urea, mg/dL	N.A.	1695.56 ± 420.12	2752.07 ± 844.13	9651.15 ± 1459.53 ***^,##^
GFR, ml/min/100 g BW	N.A.	0.20 ± 0.04	0.24 ± 0.05	0.17 ± 0.04

Blood and urine samples were collected 24 h after the final injections. Data are presented as mean ± SEM (n = 6 per group). Asterisks indicate statistically significant differences compared with the control group: * *p* < 0.05, ** *p* < 0.01, *** *p* < 0.001. Sharps indicate significant differences between the nGO and nGO-PEG-treated groups: ^##^ *p* < 0.01. ^1^ Biochemical blood parameters of male 10-week old Long Evans RjOrl:LE rats provided by Janvier Labs (Saint-Berthevin, France) [19]. N.A.—not available.

**Table 4:** Hematological parameters of the control group and groups treated with nGO and nGO-PEG nanoparticles.

Parameter	Reference Ranges ^1^	Control	nGO	nGO-PEG
WBC, ×10^9^/L	8.7 ± 1.8	4.94 ± 0.17	3.65 ± 0.65	3.93 ± 0.12 **
RBC, ×10^12^/L	8.2 ± 0.7	7.11 ± 0.11	7.38 ± 0.35	6.11 ± 0.08 ^##^
HGB, g/L	154 ± 11.0	143.80 ± 2.96	134.67 ± 7.46	119.60 ± 1.60 *
HCT, %	52.0 ± 5.0	42.42 ± 0.81	42.73 ± 2.06	34.78 ± 0.66 **^,##^
MCV, fL	63.0 ± 1.0	61.07 ± 0.41	57.95 ± 0.34 ***	56.90 ± 0.26 ***
MCH, pg	18.8 ± 0.4	18.63 ± 1.53	18.25 ± 0.42 *	19.43 ± 0.25
MCHC, g/L	300.0 ± 10.0	305.00 ± 24.82	315.17 ± 8.38	341.33 ± 5.15 ^##^
Platelet, ×10^9^/L	716 ± 203	159.20 ± 62.72	75.40 ± 11.10	95.80 ± 33.17

Blood samples were collected 24 h after the final injections. Data are presented as mean ± SEM (n = 6 per group). Asterisks indicate significant differences from the control: * *p* < 0.05, ** *p* < 0.01, *** *p* < 0.001. Sharps indicate significant differences between the nGO and nGO-PEG-treated groups: ^##^ *p* < 0.01. WBC—white blood cell count; RBC—red blood cell count; HGB—hemoglobin; HCT—hematocrit; MCV—mean corpuscular volume; MCH—mean corpuscular hemoglobin; MCHC—mean corpuscular hemoglobin concentration; PLT—platelet count. ^1^ Hematological parameters of male 10-week old Long Evans RjOrl:LE rats provided by Janvier Labs (Saint-Berthevin, France) [19].

## Data Availability

The data supporting this study’s findings are available from the corresponding authors, M.K.-M. and M.C., upon reasonable request.

## Supplementary Materials

The following supporting information can be downloaded at: https://www.mdpi.com/article/10.3390/ijms27125522/s1.

## Abbreviations

The following abbreviations are used in this manuscript:

ALT	alanine aminotransferase
AST	aspartate aminotransferase
BUN	blood urea nitrogen
BW	body weight
CK	creatine kinase
GFR	glomerular filtration rate
HCT	hematocrit
HGB	hemoglobin
LDH	lactate dehydrogenase
MCH	mean corpuscular hemoglobin
MCHC	mean corpuscular hemoglobin concentration
MCV	mean corpuscular volume
NP	nanoparticle
nGO	nanosized graphene oxide
nGO-PEG	nanosized polyethylene glycol-modified graphene oxide
PEG	polyethylene glycol
PLT	platelet count
RBC	red blood cell count
RES	reticuloendothelial system
SEM	standard error of the mean
WBC	white blood cell count
