# Supplementary material for: Systemic Effects of Repeated Intraperitoneal Application of Graphene Oxide and Polyethylene Glycol-Functionalized Graphene Oxide Nanoparticles in Long Evans Male Rats
Source: Int J Mol Sci. 2026 Jun 18;27(12):5522. doi: 10.3390/ijms27125522 (PMC13299417; doi:10.3390/ijms27125522)
Supplement: Supplementary file 1 [file ijms-27-05522-s001.zip › ijms-4361560-supplementary.pdf]

Supplementary Materials

Table S1. Urinary parameters of the control group and groups injected with nGO and nGO-PEG nanoparticles measured by test strips before and after treatment.

**Parameter**		LEU, cell/µL		KET, mmol/L		URO, µmol/L		BIL, µmol/L		PRO, g/L		GLU, mmol/L		pH	
**Group**	**№ animal**	**BT**	**AT**	**BT**	**AT**	**BT**	**AT**	**BT**	**AT**	**BT**	**AT**	**BT**	**AT**	**BT**	**AT**
**Control**	1	Neg	Neg	Neg	Neg	Normal	Normal	Neg	Neg	0.3	> 3.0	Neg	Neg	7.0	6.0
	2	15	15	Neg	0.5	Normal	Normal	Neg	Neg	Neg	> 3.0	Neg	Neg	7.0	6.0
	3	15	Neg	Neg	Neg	Normal	Normal	Neg	Neg	1.0	> 3.0	Neg	Neg	7.0	6.0
	4	15	Neg	Neg	Neg	Normal	Normal	Neg	Neg	0.3	1.0	Neg	Neg	7.0	6.5
	5	15	15	Neg	Neg	Normal	Normal	8.6	Neg	0.3	1.0	Neg	Neg	7.0	6.5
	6	Neg	Neg	Neg	Neg	Normal	Normal	Neg	Neg	0.3	0.3	Neg	Neg	7.0	6.5
**nGO**	1	15	15	Neg	0.5	Normal	Normal	Neg	Neg	0.3	> 3.0	Neg	Neg	7.0	6.0
	2	Neg	15	Neg	0.5	Normal	33.0	Neg	Neg	0.3	> 3.0	Neg	Neg	6.5	6.0
	3	Neg	15	Neg	0.5	Normal	Normal	Neg	Neg	0.15	1.0	Neg	Neg	6.5	6.0
	4	Neg	15	Neg	Neg	Normal	Normal	Neg	Neg	0.3	1.0	Neg	Neg	6.5	6.0
	5	Neg	15	Neg	0.5	Normal	33.0	Neg	Neg	0.15	> 3.0	Neg	Neg	7.0	6.0
	6	Neg	Neg	Neg	0.5	Normal	33.0	Neg	Neg	1.0	> 3.0	Neg	Neg	6.5	6.0
**nGO-PEG**	1	Neg	15	Neg	0.5	Normal	33.0	Neg	Neg	0.15	> 3.0	Neg	Neg	7.0	6.0
	2	Neg	15	Neg	0.5	Normal	Normal	Neg	Neg	0.15	1.0	Neg	Neg	7.0	6.0
	3	Neg	Neg	Neg	0.5	Normal	33.0	Neg	Neg	0.3	> 3.0	Neg	Neg	6.5	6.0
	4	Neg	Neg	Neg	0.5	Normal	33.0	Neg	Neg	0.3	> 3.0	Neg	Neg	7.0	6.0
	5	Neg	Neg	Neg	Neg	Normal	33.0	Neg	Neg	0.3	> 3.0	Neg	Neg	7.0	6.0
	6	15	Neg	Neg	0.5	Normal	33.0	8.6	Neg	1.0	> 3.0	Neg	2.8	6.0	6.0

Urinary samples were collected one day before the beginning of the treatment (BT) and 24 hours after the last injections (AT). LEU – leukocytes, KET – ketone, URO – urobilinogen, BIL – bilirubin, PRO – protein, GLU – glucose.
